# Consensus Between Radiologists, Specialists in Internal Medicine, and AI Software on Chest X-Rays in a Hospital-at-Home Service: Prospective Observational Study

**DOI:** 10.2196/55916

**Published:** 2024-12-24

**Authors:** Eitan Grossbard, Yehonatan Marziano, Adam Sharabi, Eliyahu Abutbul, Aya Berman, Reut Kassif-Lerner, Galia Barkai, Hila Hakim, Gad Segal

**Affiliations:** 1 Faculty of Medicine, University of Nicosia, Nicosia, Cyprus; 2 Dan Petah-Tikvah District at Clalit Health Services, Petah-Tikvah, Israel; 3 Chaim Sheba Medical Center, Faculty of Medicine, Tel-Aviv University, Sheba road 2, Ramat Gan, 555710, Israel, 972 526669580; 4 Department of Pediatric Intensive Care, The Edmond and Lily Safra Children's Hospital, Sheba Medical Center, Ramat Gan, Israel; 5 BEYOND Virtual Hospital, Ramat Gan, Israel; 6 Sheba Medical Center Education Authority, Ramat Gan, Israel

**Keywords:** chest x-ray, hospital-at-home, telemedicine, artificial intelligence, kappa, x-ray, home hospitalization, clinical data, chest, implementation, comparative analysis, radiologist, AI

## Abstract

**Background:**

Home hospitalization is a care modality growing in popularity worldwide. Telemedicine-driven hospital-at-home (HAH) services could replace traditional hospital departments for selected patients. Chest x-rays typically serve as a key diagnostic tool in such cases.

**Objective:**

The implementation, analysis, and clinical assimilation of chest x-rays into an HAH service has not been described yet. Our objective is to introduce this essential information to the realm of HAH services for the first time worldwide.

**Methods:**

The study involved a prospective follow-up, description, and analysis of the HAH patient population who underwent chest x-rays at home. A comparative analysis was performed to evaluate the level of agreement among three interpretation modalities: a radiologist, a specialist in internal medicine, and a designated artificial intelligence (AI) algorithm.

**Results:**

Between February 2021 and May 2023, 300 chest radiographs were performed at the homes of 260 patients, with the median age being 78 (IQR 65‐87) years. The most frequent underlying morbidity was cardiovascular disease (n=185, 71.2%). Of the x-rays, 286 (95.3%) were interpreted by a specialist in internal medicine, 29 (9.7%) by a specialized radiologist, and 95 (31.7%) by the AI software. The overall raw agreement level among these three modalities exceeded 90%. The consensus level evaluated using the Cohen κ coefficient showed substantial agreement (κ=0.65) and moderate agreement (κ=0.49) between the specialist in internal medicine and the radiologist, and between the specialist in internal medicine and the AI software, respectively.

**Conclusions:**

Chest x-rays play a crucial role in the HAH setting. Rapid and reliable interpretation of these x-rays is essential for determining whether a patient requires transfer back to in-hospital surveillance. Our comparative results showed that interpretation by an experienced specialist in internal medicine demonstrates a significant level of consensus with that of the radiologists. However, AI algorithm-based interpretation needs to be further developed and revalidated prior to clinical applications.

## Introduction

### Home Hospitalization in the Post–COVID-19 Era

Home hospitalization has gained popularity in recent years as a cost-effective alternative to in-hospital care, especially for patients with chronic conditions or those who require short-term medical attention. This approach offers numerous advantages such as improved patient outcomes, reduced health care costs, and increased patient satisfaction [1-4]. Nevertheless, hospital-at-home (HAH) programs are still considered experimental and have yet to gain the appropriate adoption by physicians and reimbursement organizations worldwide. They warrant comprehensive plans for further research and development [5,6]. In 2020, as part of our reorganization and development of a flexibility-design program during the COVID-19 pandemic [7], we established a unique HAH service known as Sheba Beyond. This virtual hospitalization arm of our medical center operating as part of the tertiary hospital, has served a diverse population of more than 1000 patients since its establishment, experiencing rapid acceleration in usage post–COVID-19 pandemic [8].

### Telemedicine-Based HAH Service

While a traditional hospital is still the standard setting for managing acute conditions, it poses challenges for high-risk patients, especially older adults who are vulnerable to iatrogenic conditions such as hospital-acquired pneumonia [9,10]. Changes in the environment, such as the transportation of patients for in-hospital imaging examinations might worsen their overall frail condition. To create a viable alternative to in-hospital services, we developed an HAH service that ensures both efficacy and safety for treating patients with acute illness. This required us to commit to two key pillars of service. First, attending physicians must be experienced internal medicine specialists who provide care for their patients from within our medical center, relying on telemedicine platforms. Second, over the past 3 years, this approach has proven effective and safe, and was supported by a continuous effort to explore and validate all our telemedicine-based services [11-13].

### Chest X-Ray as Part of the Management of Patients With Acute Illness

The basic chest x-ray serves as the cornerstone for the initial diagnosis of many acute conditions, mainly for patients presenting with acute shortness of breath. Nevertheless, its interpretation is subject to a high level of interobserver variability [14]. The application of chest x-rays in patients with suspected community-acquired pneumonia is commonly practiced, although various guidelines and reviews offer differing approaches toward this practice and interpretive value [15]. As part of our decision to enhance our HAH service for patients who were acutely ill using a comprehensive safety investigation and treatment plan, we decided to include home-based, mobile chest x-rays in routine HAH admissions.

At-home x-ray services allow carers to avoid unnecessary and potentially harmful transfers from patients’ homes and enable the diagnoses of patients who might otherwise remain unexamined such as those who are bed-bound or too ill to be transferred to the hospital. In such cases, it may be best to carry out x-ray examinations at the patient’s home [16]. Considering that x-ray examination is the most frequently conducted imaging procedure during hospitalization [17,18], and due to the potential to provide hospital-level care at home [19], this study aimed to describe, characterize, and analyze our unique experience with diagnostic imaging within the HAH setting.

## Methods

### Research Setting of the Current Study

We performed a prospective observational study to examine a population of HAH patients who underwent chest x-rays at home and to assess the level of agreement among three interpretation modalities. The study included consecutive patients admitted to the Sheba Beyond virtual hospital HAH service between February 2021 and May 2023. All patients aged 18 years and older who underwent chest x-ray examinations during their hospitalization were included. These examinations were performed as part of the clinical routine and no patient was excluded, as per the research criteria.

### Ethical Considerations

This study was approved by the Sheba Medical Center institutional review board (0345‐23- SMC), which waived the need for informed consent as there was no intervention involved or deviation from the standard patient care, and all chest x-ray interpretations were analyzed on an ad hoc basis.

During the entire data mining and analysis process, complete confidentiality of participants’ data was maintained; the deidentified data was only accessible to the research team. A coded list of patients was available exclusively to the principal investigator, as required by the institutional review board. The study participants were not provided any compensation, and the final manuscript does not include any potentially identifiable patient data.

### At-Home Chest X-Rays as Part of Our HAH Service

As part of the initial investigation of HAH patients, a trained technician conducted the chest x-ray examinations using a mobile device at the patient’s home. The anteroposterior projection. The at-home chest x-rays were performed using the Fujifilm portable x-ray unit FDR XAIR (Fujifilm UK Ltd, Bedford, UK), a compact device, measuring 301×257×144 mm and weighing approximately 3.5 kg. The unit features a tube voltage range of 50-90 kV and a tube current time range of 0.20-2.50 mAs. Furthermore, the device operates on a lithium polymer battery (11.1 V, 1450 mAh), making it ideal to use in the HAH setting where an external power source may not always be readily available by the patient’s bedside. Additional accessory equipment included a detector, holding device, and laptop computer.

Initial interpretation of HAH x-rays was always performed by the attending physician, who was an experienced internal medicine specialist. These interpretations were documented in the patients’ electronic medical records. In some cases, additional interpretations were performed by a specialized radiologist. However, they were conducted later during or after the HAH period and were not included in the patients’ routine clinical investigations, which is the standard practice for in-hospital patients as well. Furthermore, interpretation was also provided by artificial intelligence (AI) software. While this interpretation was performed by default, it was not considered reliable or essential for clinical purposes. The AI software used in this study was developed by Aidoc, a Food and Drug Administration–approved health care–AI company that markets several types of health care–associated AI software, including algorithms that analyze radiological images. This software retrospectively processed radiographs and provided interpretations as well as highlighted potentially pathological areas in the image.

### Statistical Analysis

Continuous variables were presented as mean (SD) for normally distributed data or as median (IQR) for skewed data. We determined the normality of variables by using Anderson-Darling and Shapiro-Wilk tests. Categorical variables were presented as frequencies (%). All analyses were performed using R software (version 4.1.0; R Foundation for Statistical Computing).

We performed a comparative analysis, evaluating the level of agreement across three interpretation modalities: (1) a physician specializing in imaging (radiologist), (2) the attending physician, (a specialist in internal medicine), and (3) a designated AI algorithm. Initially, we compared the agreement between the specialist in internal medicine and the radiologist, followed by assessing the agreement between the specialist in internal medicine and the AI software. Consequently, we first identified the interpretation given to each x-ray image. Some examinations included multiple findings, such as both lobar consolidation and pleural effusion. When comparing two interpreters, we considered all x-ray images with interpretations from both and recorded how often each interpreter agreed or disagreed on each pathology (ie, whether both interpretations indicated “yes” or “no” for a specific finding). To measure the level of consensus between interpreters, we calculated the Cohen κ coefficient for interrater reliability, which subtracts the likelihood of random agreement from the overall agreement [20,21], to classify the level of agreement. Table S1 in Multimedia Appendix 1 summarizes the possible results for Cohen κ coefficient.

## Results

The study included a total of 260 patients who were hospitalized over 27 months at the Sheba-Beyond HAH service, during which a total of 300 chest x-ray examinations were performed. Some patients were admitted more than once and underwent several imaging sessions. The median age of the patients was 74 (IQR 62-87) years. Of the 260 patients, 55% (n=143) of the patients were women. The median BMI was 25.86 (IQR 22.5-32.1). The median length of an HAH stay was 3 (IQR 2-4) days. Cardiovascular disease was the most prevalent underlying morbidity in our patients (n=185, 71.2%). Table 1 presents the varied distribution of background diagnoses within our study cohort.

Upon admission, the patients were categorized with a primary working diagnosis, with some being modified at discharge, while certain patients had multiple diagnoses. The most common acute medical conditions during hospitalization were infectious (78%), respiratory (10%), gastrointestinal/genitourinary (5%), and cardiovascular (5%) disease. The distribution of these diagnoses is presented in Table 2.

As shown in Tables1  2, the most frequent acute diagnoses were related to infectious diseases affecting the respiratory system and the genitourinary and gastrointestinal systems, predominantly in patients with underlying cardiovascular disease. The internal medicine specialist interpreted the majority (n=286, 95.3%) of the 300 x-rays. About 10% (n=29) of the x-rays were interpreted by a radiologist and 31.7% (n=95) by the AI software. Some of the x-ray images showed multiple findings. Table 3 displays the distribution of different pathologies according to the interpreters.

We evaluated the consensus level between interpreters regarding the most common 16 x-ray diagnoses. The level of raw agreement between the specialist in internal medicine and the radiologist was 95.5%, while that between the specialist in internal medicine and the AI software was 93%. The consensus level evaluated using the Cohen κ coefficient showed substantial (κ=0.65) and moderate agreement (κ=0.49) between the specialist in internal medicine and a radiologist, and between the specialist in internal medicine and the AI, respectively. The comparison of consensus levels is presented in Table 4.

**Table 1.** Distribution of background diagnoses and chronic diseases within the study cohort.

Disease category	Patients (N=260), n (%)
Cardiovascular disease	185 (71.2)
Endocrine disease	88 (33.8)
Gastrointestinal/genitourinary disease	80 (30.8)
Oncological/immunodeficient disease	78 (30.0)
Rheumatological/musculoskeletal disease	60 (23.1)
Neurological disease	54 (20.8)
Other diseases	34 (13.1)
Respiratory disease	27 (10.4)

**Table 2.** Distribution of acute patient diagnoses at admission and discharge.

Diagnosis	Upon admission (N=260), n (%)	At discharge (N=260), n (%)
Infectious disease	202 (77.7)	208 (80.0)
Respiratory disease	21 (8.1)	26 (10.0)
Gastrointestinal/genitourinary disease	13 (5.0)	26 (10.0)
Cardiovascular disease	8 (3.1)	16 (6.2)
Rheumatological/musculoskeletal disease	3 (1.2)	3 (1.2)
Neurological/psychiatric disease	3 (1.2)	3 (1.2)
Hematological disease	0 (0)	16 (6.2)
Endocrine, metabolic, and electrolytic disorders	0 (0)	18 (6.9)

**Table 3.** Distribution of x-ray interpretations according to a radiologist, internal medicine specialist, and artificial intelligence software.

Diagnosis	Pathology interpreted by an internal medicine specialist (n=286), n (%)	Pathology interpreted by a radiologist (n=29), n (%)	Pathology interpreted by artificial intelligence software (n=95), n (%)
Normal study	157 (54.9)	13 (44.8)	42 (44.2)
Infiltrate/consolidation	60 (21.0)	13 (44.8)	39 (41.1)
Unclear shadow	28 (9.8)	3 (10.3)	0 (0.0)
Pleural effusion	20 (7.0)	6 (20.7)	13 (13.7)
Lung congestion	11 (3.8)	0 (0.0)	0 (0.0)
Mediastinal widening/cardiomegaly/lymphadenopathy	11 (3.8)	3 (10.3)	16 (16.8)
Space-occupying lesion	28 (9.8)	0 (0.0)	0 (0.0)
Atelectasis	0 (0.0)	0 (0.0)	9 (9.5)
Metastases/nodules	0 (0.0)	0 (0.0)	2 (2.1)
Aortic calcifications	0 (0.0)	3 (10.3)	0 (0.0)
Diaphragmatic hernia	0 (0.0)	3 (10.3)	0 (0.0)
Pneumothorax	0 (0.0)	0 (0.0)	2 (2.1)

**Table 4.** Level of agreement between different interpreters.

Modalities	Raw agreement (%)	Cohen κ coefficient	Strength of agreement
Internal medicine specialist and radiologist	95.5	0.65	Substantial
Internal medicine specialist and artificial intelligence software	93	0.49	Moderate

## Discussion

The results of this study show that using three modalities for HAH-based x-ray interpretations yielded substantial agreement between radiologists and specialists in internal medicine, while a moderate agreement was achieved between interpretations of the internal medicine specialist and the AI algorithm.

### Study Population and Contribution of Chest X-Rays to Diagnosis

Assimilation of new technologies into HAH services should be encouraged and thoroughly validated before being recommended for inclusion in relevant guidelines. This principle guided our prior research [11-13] and publications, and it also forms the rationale for this study which aims to validate the integration of mobile chest x-rays into our HAH service.

The patient population in this study represents the typical demographic of hospitalized patients seen in internal medicine departments, predominantly older individuals (median age 74 years) with an underlying cardiovascular morbidity (71%) and equal gender distribution (55% were women). The primary working or hospitalization diagnosis was respiratory infection, with respiratory and infectious conditions being the most common diagnoses, both at admission and upon discharge. Interestingly, the most common interpretation of the chest x-rays of these patients was labeled as a normal study, with all three modalities providing comparable values (internal medicine specialist: 55%; radiologist: 44%; and AI software: 45%). The second most common interpretation of their chest x-rays was ascertained to be pulmonary infection, with pulmonary consolidation diagnosed in 21%, 44%, and 41% of cases across internal medicine specialists, radiologists, and AI software, respectfully.

From these findings, it can be concluded that the majority of our patients were suspected to have pneumonia, and therefore, a routine chest x-ray examination upon admission was plausible. Nevertheless, it is observed that the impact of the findings of chest x-rays on altering the initial admission diagnosis (documented prior to chest x-rays) was minimal. This could be due to diagnoses frequently being based on patients’ anamnesis and medical history, which is consistent with in-hospital stays.

### Level of Consensus Between Modalities

Examining the raw data presented in Table 3 reveals that among patients with the most frequent diagnoses (normal study, pulmonary consolidation, and pulmonary infiltrate), the radiologist and the AI algorithm exhibited greater consensus compared to the specialist in internal medicine. This can be due to both (the radiologist and AI) having little to no background information about the patients’ chief complaints and anamnesis. This reinforces the notion that the diagnosis of pulmonary infections relies more heavily on history-taking rather than chest imaging. Future AI modalities engaged in x-ray interpretation should have clinical background and patient data integrated into them to achieve better results.

The raw level of consensus between all three modalities was very high. This alone may create the impression of a unanimous diagnosis between both physicians and AI. Nevertheless, the role of Cohen κ coefficient is to reveal the true consensus beyond the scope of default accidental agreement. Analyzing the κ values achieved in our study, it is not surprising that both the physicians achieved a substantial level of consensus (κ=0.65), while the AI and the specialist in internal medicine achieved only a moderate level of consensus (κ=0.49). This indicates that AI is still in its early stages regarding the interpretation of chest x-rays, and to our best knowledge, it is not yet regulated as an official diagnostic modality worldwide.

It is important to note that our κ values should not be viewed as surprisingly low. In a 2006 study, Novack et al [22] showed low levels of consensus regarding chest radiography interpretations between a radiologist, pulmonologist, and infectious disease specialist in patients with suspected pneumonia. The κ values ranged from 0.09 to 0.44. However, these discrepancies did not affect the clinical outcomes.

Previous studies have investigated the use of AI algorithms for similar purposes. Rudolph et al [23] found that AI outperformed nonradiology residents in interpreting chest radiographs while matching the performance levels of radiology residents. However, their study did not explore the HAH settings and used consensus-level measurements other than the κ coefficient. Wu et al [24] employed the κ coefficient to measure the consensus level between a trained AI algorithm and third-year radiology residents, reporting a κ value of 0.585. Similar to previous research, their study did not focus on the HAH settings nor compared the results with the attending senior physician’s interpretations. Sridharan et al [25] succeeded in assimilating AI software designed to interpret chest x-rays in a high-turnover triage environment. In their clinical study which compared the evaluations of 43 radiologists who were blinded to the AI interpretations, they achieved a high level of accuracy of AI interpretations with sensitivity, specificity, and area under the curve consistently above 84%. However, this study also did not include HAH patients. Other studies have evaluated the diagnostic yield of AI-based chest x-rays, specifically for the examination and prognostication of patients with COVID-19 [26-28].

### Study Limitations

This was a single-center study involving a limited number of patients and physicians, potentially contributing to an interpretation bias. Additionally, we used a specific technology for mobile chest x-ray examinations and a designated AI algorithm for interpretation, which could potentially introduce a bias in our results. It is logical to assume that using a larger number of patients and multiple image acquisitions and interpretation modalities would yield more robust results and conclusions. Future research should concentrate on both the expansion of x-rays in the HAH environments and the assimilation of AI-based algorithms for the interpretation of x-rays across a larger patient population.

### Conclusions

As the global demand for expanding and developing infrastructure of HAH services with the aim of replacing in-hospital stays of patients who are acutely ill keeps growing, the use of mobile chest x-rays at patients’ homes is a feasible option.

Given that respiratory infections continue to be a leading cause of acute hospitalization, we anticipate a continuous need for mobile chest x-rays in HAH services for such patients. However, chest x-rays are less informative compared to a detailed patient history and physical examination. This limitation applies equally to both in-hospital and HAH settings.

The application of AI algorithms for routine clinical interpretation of chest x-rays should remain within the domain of experimental diagnostics until further research demonstrates higher levels of consensus, ideally surpassing those observed in this study.

Future research should concentrate on three key directions as reflected by our study results: (1) expanding the HAH modality to better understand and improve these essential services, (2) broadening the use of training and validation of AI-based algorithms in the field of x-ray interpretation, and (3) incorporating clinical data into the specific AI algorithms to improve consensus between physician and AI-based x-ray interpretations.

## Supplementary material

10.2196/55916 Multimedia Appendix 1 Level of agreement according to the Cohen κ coefficient.

## Abbreviations

AI: artificial intelligence
HAH: hospital-at-home
