# Supplementary material for: Consensus Between Radiologists, Specialists in Internal Medicine, and AI Software on Chest X-Rays in a Hospital-at-Home Service: Prospective Observational Study
Source: JMIR Form Res. 2024 Dec 24;8:e55916. doi: 10.2196/55916 (PMC11693780; doi:10.2196/55916)
Supplement: Multimedia Appendix 1 [file formative-v8-e55916-s001.docx]

*Table 1: Level of agreement according to the Cohens’ Kappa Coefficient*

| **Cohen's Kappa** | **Interpretation** |
| --- | --- |
| < 0.00 | Poor |
| 0.00-0.20 | Slight |
| 0.21-0.40 | Fair |
| 0.41-0.60 | Moderate |
| 0.61-0.80 | Substantial |
| 0.81-1.00 | Almost perfect |
